# Microfinance institutions failure prediction in emerging countries, a machine learning approach

**DOI:** 10.1371/journal.pone.0321989

**Published:** 2025-04-24

**Authors:** Yvan J. Garcia-Lopez, Patricia Henostroza Marquez, Nicolas Nuñez Morales

**Affiliations:** 1 CENTRUM Católica Graduate Business School (CCGBS), Lima, Peru; 2 Pontificia Universidad Católica del Perú (PUCP), Lima, Peru; IIM Sambalpur, INDIA

## Abstract

This study is about what matters: predicting when microfinance institutions might fail, especially in places where financial stability is closely linked to economic inclusion. The challenge? Creating something practical and usable. The Adjusted Gross Granular Model (ARGM) model comes here. It combines clever techniques, such as granular computing and machine learning, to handle messy and imbalanced data, ensuring that the model is not just a theoretical concept but a practical tool that can be used in the real world.Data from 56 financial institutions in Peru was analyzed over almost a decade (2014–2023). The results were quite promising. The model detected risks with nearly 90% accuracy in detecting failures and was right more than 95% of the time in identifying safe institutions. But what does this mean in practice? It was tested and flagged six institutions (20% of the total) as high risk. This tool’s impact on emerging markets would be very significant. Financial regulators could act in advance with this model, potentially preventing financial disasters. This is not just a theoretical exercise but a practical solution to a pressing problem in these markets, where every failure has domino effects on small businesses and clients in local communities, who may see their life savings affected and lost due to the failure of these institutions. Ultimately, this research is not just about a machine learning model or using statistics to evaluate results. It is about giving regulators and supervisors of financial institutions a tool they can rely on to help them take action before it is too late when microfinance institutions get into bad financial shape and to make immediate decisions in the event of a possible collapse.

## Introduction

Throughout this research, it becomes abundantly clear that microfinance institutions, or MFIs, have an important job helping in places where money and resources are scarce. They fill a surprisingly large gap left by traditional banks that ignore smaller, often overlooked businesses [1]. By offering loans, savings options, insurance, and even lessons on managing finances, MFIs sincerely intend to improve things in communities that need a boost, making things fairer regarding who has money and who doesn’t [2].

They are focused on creating more jobs, boosting businesses, and ensuring more people have a fair chance at growth, but while MFIs are doing some pretty important work, they are still lacking in convincing new clients with their products [3]. Their efforts to reach out to clients who don’t normally get enough attention from mainstream banks means that they are heavily involved in places where the financial situation can change very quickly [4]. This is a major problem because if an MFI fails, it is not simply a mistake. It could lead to serious problems, such as people losing their savings immediately or making it even harder for everyone to have a fair shot at economic opportunities in the future [5]. Now, you understand why MFIs are a big deal. In theory, but not necessarily in fact, they are supposed to break down various barriers to make economies stronger and more inclusive.

That part is key: they are in this to fight the monetary inequality that keeps many people in poverty [3]. Here, in this situation, that noble goal is in trouble. The problem is that the things that make MFIs able to help people also put them at risk of collapsing if things go wrong, particularly during tough economic times.

This fear is not limited to a few people losing out; it is mostly about the possibility of implementing strategies that will improve the lives of many people and even entire countries. The research is focused on building an intelligent and informed predictive model just for microfinance [6].

On the other hand, the usual ways of guessing when financial places might fail do not capture all the special aspects of microfinance in places where the economy is just starting since understanding when these institutions are likely to get into trouble is vitally important to keep everyone’s money safe, fully verify that the economy remains on track, and not to lose all the remarkably positive contributions that microfinance has made, we need better analytical tools.

Something needs to be created that could do the job. This proposal looks at specific things that warn us if a microfinance institution might be in trouble. Giving these more accurate tools to individuals or people who need to be on the lookout for financial risks means they can intervene earlier and more effectively. In addition, the work helps to understand how to keep microfinance strong, which is an important issue in helping poorer areas grow economically without leaving anyone behind.

In doing all this, the study significantly changes the way the concentrated environment, or world, of microfinance is understood and protected. This is not just about theory; it is primarily about ensuring that these banks and microfinance institutions can continue to do their work without unexpectedly failing completely.

## Theoretical and empirical literature review

Specifically, financial problems are rapidly aggravated by difficulties suffered by institutions, exacerbating macroeconomic deterioration. When banks are in trouble, things spiral out of control: jobs disappear, loans become extremely difficult to obtain, and everything becomes complete chaos [7]. Because of all the chaos mentioned, regulatory institutions decided to establish stricter rules to prevent banks from losing financial soundness and collapsing so easily [8]. Furthermore, predicting whether a bank or microfinance institution will fail has become much more complicated over the years [9,10]

The evolution of financial failure prediction models reflects the progressive development of increasingly sophisticated analytical techniques. From Altman’s seminal work with discriminant analysis [11], which established the importance of financial ratios in failure prediction, progress was made towards Logit and Probit models that improved predictive capacity. In parallel, the CAMEL model [12] emerged as a comprehensive framework for assessing the financial health of banking institutions, proving particularly effective in emerging markets [13] despite its limitations in capturing systemic risk [14].

Since the 1990s, computers have become very smart with machine learning, a technology that learns through patterns. They started using neural networks [15,16],[a special way computers learn, and decision trees [17] to improve the prediction of bank errors.

These types of algorithms became very good [18]. Still, the problem is that sometimes, these algorithms can be difficult to understand or even receive the data wrong due to hidden problems in their learning [18]. At its most basic level, we have become experts at guessing when banks might get it wrong, but thinking about the intricacies of what these technological tools are thinking? That’s another story [9].

To start and get familiar, we delve into what everyone has been talking about in the concentrated environment (or world) of learning to control when financial failures can occur. We are examining a trio of indicators: financial ratios [19], macroeconomic variables [8], and capital market indicators [20]. In emerging markets, where many microfinance institutions are not publicly traded, predictive models have focused primarily on financial ratios, combining traditional metrics from the Altman model [11]with financial sector-specific indicators such as those from the CAMEL framework [12].

Recent studies have demonstrated the effectiveness of this approach in similar contexts [21,22], particularly when the indicators are adapted to the specific characteristics of the local market [23].

They’ve mixed in outdated material from Altman’s Z-score [24] playbook with some components honed specifically for the money-lending public, courtesy of the CAMEL checklist. It’s worked wonders, especially if they tweak it to fit the local scene perfectly. Moving on to something new, the details of avoiding a big failure in these hot markets are getting a major makeover. They’ve gone from looking at Altman’s Z-score treasure box straight into the intellectual realm of computer learning on the fly – machine learning is stealing the show.

We can easily see that using a combination of Random Forest and Gradient Boosting [25], after some SMOTE preprocessing, sharpens the crystal ball picture quite a bit, i.e., it is more accurate [26]. But there is more: diving into deeper regions with deep learning [27] and selecting the best features with XGBoost [28] is a very high-performing model.

The selected financial ratios (S1 Appendix) are grouped into five categories following the CAMEL framework: Capital Adequacy (8 ratios), Asset Quality (11 ratios), Management Capability (5 ratios), Profitability (4 ratios), and Liquidity (4 ratios). This selection integrates traditional Altman indicators with metrics specific to the microfinance sector. Although this model has limitations inherent to financial statements, the reliability of these reports means that the selected variables have adequate predictive power [29,30].

## Materials and methods

### Study sample

The Peruvian microfinance sector represents a relevant case study due to its high level of development and its pioneering role in the region [new high-impact reference on microfinance in emerging markets]. This allows for evaluating the effectiveness of predictive models in a mature market with characteristics of emerging economies.

### Data processing

Table 1 displays Peruvian financial institutions’ status between 2014 and 2023. Many of them were affected by the economic crises, changes in the game’s rules by the governments in power, and the Covid-19 pandemic. All samples are described by 39 attributes of financial ratios evaluated. All data were grouped into a single data set to be assessed by the model (S1 Database). Each dataset analyzed by period has unbalanced characteristics (S2 Appendix). In addition, the data sets have missing values. The percentage of cases with missing values in the data set ranged from 13% to 15%. Therefore, it was challenging to preprocess the original data properly. A more detailed analysis of the data decomposition complexity was performed using the ARGM algorithm.

**Table 1:** Failure dataset description.

Data Set	N^o^ Finance Ratios (Source)	N^o^ Financial Institutions	Values	Missing Values	% Missing Values
**2014**	39	51	1891	283	14.97
**2015**	39	47	1828	291	15.92
**2016**	39	45	1754	234	13.34
**2017**	39	45	1755	229	13.05
**2018**	39	45	1755	275	15.67
**2019**	39	44	1713	271	15.82
**2020**	39	44	1755	234	13.33
**2021**	39	44	1716	256	14.92
**2022**	39	44	1714	239	13.94
**2023**	39	43	1676	228	13.60

Therefore, the financial ratios to be applied in our model were reduced to 32, eliminating those with 40% or more of missing data to ensure more accurate results in the classification model.

### Quality metrics

Inter-class imbalance is an inherent challenge in financial failure prediction [31], where failing institutions (minority class) represent a small but critical sample fraction. This imbalance affects the predictive performance of traditional machine learning models [32], requiring specific balancing techniques and more suitable evaluation metrics than conventional accuracies, such as ROC and Precision-Recall curves [33].

To attenuate class imbalance, the literature [31] identifies four important treatments: (1) machine learning algorithms that modify the learning process, (2) oversampling techniques such as SMOTE, (3) cost-sensitive methods that adjust the classification weights of the imbalanced model, and (4) ensembles of machine learning algorithms that combine multiple classifiers.

This study’s approach, which integrates SMOTE with machine learning techniques (Random Forest and Gradient Boosting), leads to a notable improvement in the predictive capacity of imbalanced data [34]. This enhancement holds significant potential for future applications. When you look at the data collected from 56 financial places in Peru, which include assorted banks and microfinance, such as the ones helping small communities from 2014 to 2023, only a small slice, 11.44% to be exact as cases, were not doing particularly well. This stark disparity underscores the necessity for specific balancing techniques, justifying this study’s approach.

The sample comprises 56 Peruvian financial institutions (including banks, microfinance, and municipal/rural financial institutions) observed during the period 2014–2023, with an imbalance index (RI) of 18.67. This asymmetry in the data required the implementation of specific assessment metrics for imbalanced data, including sensitivity, specificity, F1 score, MCC, and AUC [23].

Traditional metrics such as accuracy prove inadequate in financial failure prediction contexts, where misclassification costs are asymmetric [9]. Therefore, our analysis focuses mainly on the area under the ROC curve (AUC) and the Matthews correlation coefficient (MCC), complemented with specific metrics for unbalanced data such as sensitivity and specificity. This combination of metrics allows us to evaluate the model performance robustly, especially considering the critical cost of misclassifying a failing institution as healthy [33].

### Proposed approach: Rough granular computing modeling (RGCM)

Building upon previous research on failure prediction in financial institutions [25], we propose an enhanced approach that addresses the class imbalance problem through selective synthetic sampling [35]. Our work extends recent developments in financial credit risk assessment [36] by incorporating a granular approach using manually adjusted machine learning ensemble models to generate minority class examples in homogeneous feature spaces. This approach is particularly relevant for highly imbalanced financial datasets, where traditional methods often underperform [37].

Hence, the proposed Adjusted Rough-Granular Model (ARGM) integrates rough set theory and granular computing with ensemble techniques like Random Forest (RF) and Gradient Boosting (GB) [34,38,39]. This hybrid approach aims to improve classification performance in uncertain and high-dimensional imbalanced data scenarios while maintaining model interpretability. The ARGM consists of six key steps:

1. Model Ensemble: Combines RF and GB for robust prediction
2. Data Granules Formation: Segments data using granular computing principles
3. Granules Evaluation: Identifies uncertainty areas using rough sets
4. Processing Mode Selection: Optimizes classifier selection per granule type
5. Synthetic Sampling: Generates balanced training data using SMOTE
6. Inconsistent Sample Filtering: Removes noise through tolerance-based rough sets.

The model validation employed 5-fold cross-validation with GridSearchCV optimization, achieving accuracy scores of 98.51% and 95.52% for Random Forest and Gradient Boosting, respectively.

## Results and discussion

From the above observations, 25 symmetrical attributes suggest that most of the data are evenly distributed, which is ideal for the model being worked on in this research. Subsequently, we split the dataset using the Random Sampling technique, using 70% of the data for training and 30% for testing.

### Model performance comparison

In Table 2, the base logistic regression model’s performance on the imbalanced dataset (IR = 18.67) revealed a significant bias toward the majority class. While achieving 91.46% overall accuracy, it showed poor failure detection capability (sensitivity = 0.4444).

**Table 2:** Key performance metrics comparison.

Metric	Base Model (Test)	SMOTE-Enhanced	Improvement
**Sensitivity**	0.4444	0.9020	+0.4576
**Specificity**	0.9726	0.9608	-0.0118
**F1-Score**	0.5333	0.9293	+0.3960
**AUC**	0.7085	0.9310	+0.2225

**Fig 1:**
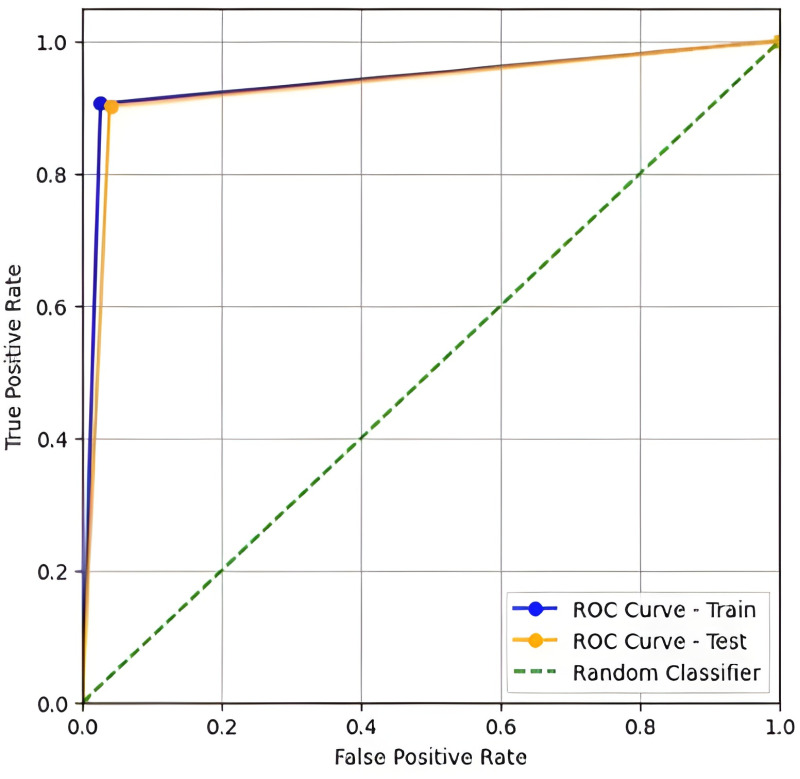
Curve ROC of the base logistic regression model.

SMOTE rebalancing dramatically improved minority class detection while maintaining high specificity. Most notably, sensitivity increased by 45.76 percentage points in the test set, with minimal specificity loss (-1.18%). The F1-Score improvement (+0.3960) and AUC gain (+0.2225) confirm the enhanced model’s superior discriminative power. (see Fig 1)

The curve ROC (Receiver Operating Characteristic) of the base logistic regression model’s performance on the imbalanced dataset (IR = 18.67) revealed a significant bias toward the majority class.

After the 32 financial ratios were prepared, the balancing processes were carried out without using the SMOTE technique. From a sample of 271 records, 189 records, representing 70%, will be taken for training, and 82 records, representing 30%, will be taken for testing, as shown in Table 3. We will construct a simple logistic regression classifier and compare the classifier’s results without SMOTE.

**Table 3:** Confusion matrix for training and test data set.

Dataset	Actual Class	Predicted Class: Healthy	Predicted Class: Failure	Total
**Training Data**	Healthy	167	0	167
	Failure	10	12	22
**Testing Data**	Healthy	71	2	73
	Failure	5	4	9

**Fig 2:**
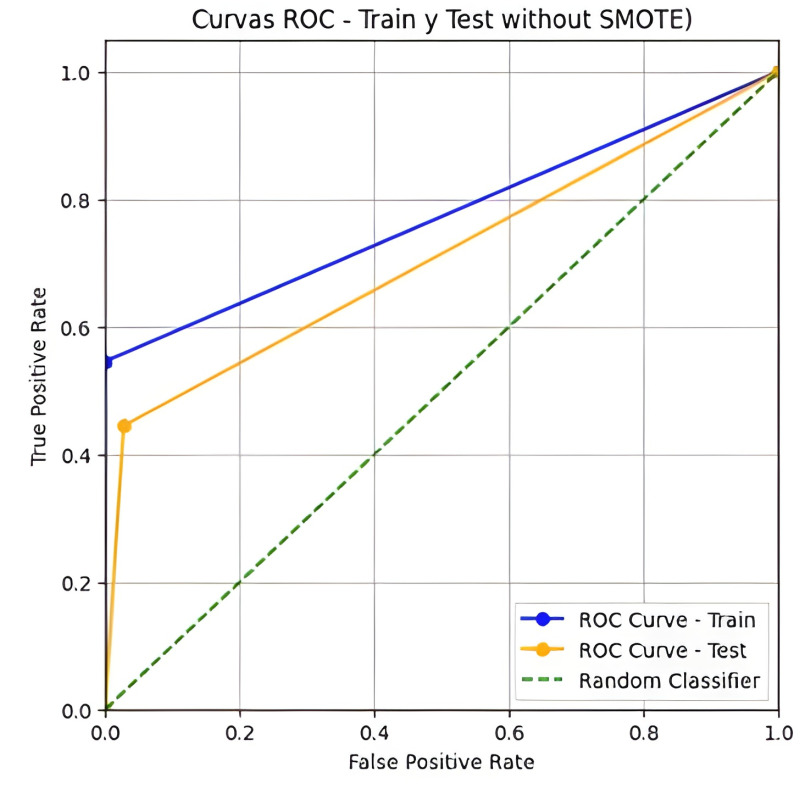
Curve ROC of the training and test dataset without SMOTE.

Table 3 shows the model’s performance in training and test sets. In the training set, with 189 records, the model correctly predicted 167 healthy institutions with no false positives. However, it misclassified ten failing institutions as healthy (false negatives). In the test set containing 82 records, the model correctly classified 71 healthy and four failing institutions. However, five failing institutions were classified as healthy (false negatives), and two healthy institutions were incorrectly classified as failing (false positives). The ROC curve shown is in Fig 2.

Initial logistic regression without SMOTE revealed limitations in handling the imbalanced dataset (IR = 18.67). While achieving high overall accuracy (91.46% in the test set), the model showed poor performance in identifying institutions at risk of failure, with a sensitivity of only 0.4444 in the test set (see Table 4).

**Table 4:** Comparison of the base model versus smote-enhanced.

	Base model		SMOTE-Enhanced	
**Metric**	**Train**	**Test**	**Train**	**Test**
**Specificity**	0.9901	0.9726	0.9741	0.9608
**Sensitivity**	0.5455	0.4444	0.9052	0.9020
**F1-Score**	0.7059	0.5333	0.9375	0.9293
**AUC**	0.7674	0.7085	0.9400	0.9310

**Fig 3:**
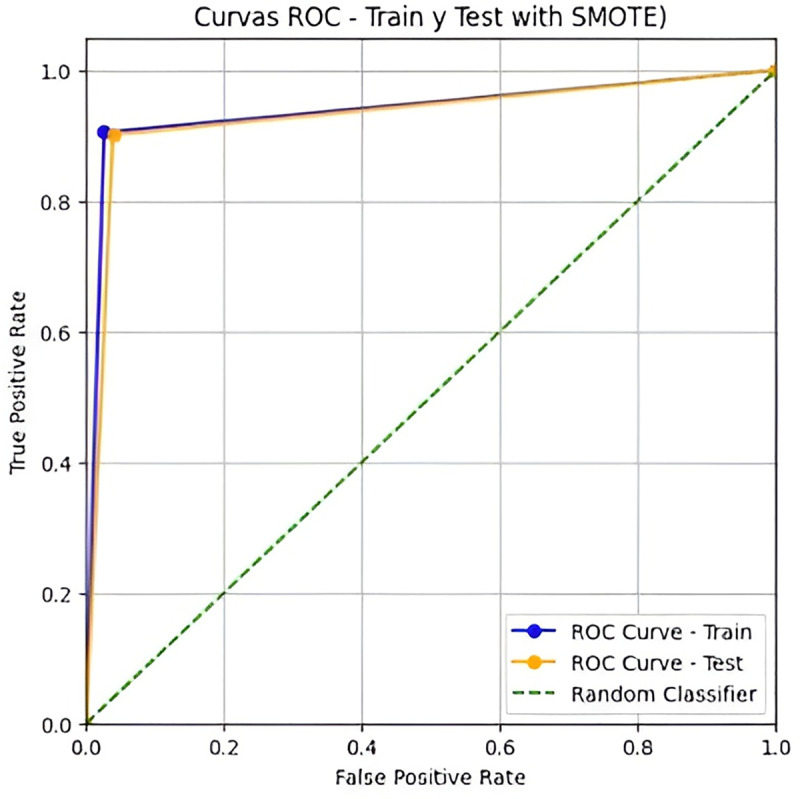
Curve ROC of training and test dataset with SMOTE.

The SMOTE-enhanced model significantly improved minority class detection while maintaining high specificity. Most notably, sensitivity increased from 0.4444 to 0.9020 in the test set, demonstrating robust generalization. The substantial improvement in F1-Score (0.5333 to 0.9293) and AUC (0.7085 to 0.9310) confirms the effectiveness of the balanced approach. Fig 3 shows the ROC curve comparison, highlighting the enhanced discriminative power of the SMOTE-based model.

Overall, the model shows good performance, but false negatives in both sets (training and testing) still represent a risk, as they might not detect institutions in danger of bankruptcy, which is critical in a financial environment.

Table 5 shows an Accuracy for the training dataset of 94.71% and 91.46% for the Accuracy testing dataset. One could say that the model has performed exceptionally well, but one should not rely on this first result; one should examine the other reported data. The model has a high specificity in both sets (0.99 and 0.9726), indicating that it identifies healthy institutions well. However, sensitivity is low, especially in the test set (0.4444), reflecting difficulties in detecting failures. Although accuracy is high in training (0.99), it decreases significantly in the test (0.6667), indicating a deterioration in performance outside of training. The drop in F1-Score and MCC suggests that model balancing needs to be improved, possibly using techniques such as SMOTE to balance the classes.

**Table 5:** Train and test classification evaluation metrics.

Metric	Base Model (Training)	Base Model (Testing)	SMOTE-Enhanced (Training)	SMOTE-Enhanced (Testing)	Improvement
**Specificity**	0.9901	0.9726	0.9741	0.9608	-0.0118
**Sensitivity**	0.5455	0.4444	0.9052	0.902	0.4576
**F1-Score**	0.7059	0.5333	0.9375	0.9293	0.396
**AUC**	0.7674	0.7085	0.94	0.931	0.2225
**Accuracy**	0.9471	0.9146	0.9397	0.9314	0.0168

With the same 32 financial ratios, a new data analysis was performed to perform a balance with the SMOTE technique. Table 6 shows the training set; the model correctly classified all instances of both classes (116 healthy and 116 failing), suggesting perfect performance. On the other hand, in the test set, the model correctly classified 51 healthy and 51 failing institutions.

**Table 6:** Confusion matrix training and test data set.

Dataset	Actual Class	Predicted Class: Healthy	Predicted Class: Failure	Total
**Training Data**	**Healthy**	113	3	116
	**Failure**	11	105	116
**Testing** **Data**	**Healthy**	49	2	51
	**Failure**	5	46	51

The data set is balanced by applying the SMOTE technique in Fig 3.

The balance achieves an Accuracy of 93.97% for the training dataset and 93.14% for the Accuracy testing dataset. To better assess the results, we will review the confusion matrix with the training and testing datasets in Table 7.

**Table 7:** Training and test classification evaluation metrics with SMOTE.

	Training dataset	Test dataset
**Specificity**	0.9741	0.9608
**Sensibility**	0.9052	0.9020
**Precision**	0.9722	0.9583
**Accuracy**	0.9397	0.9314
**F1-Score**	0.9375	0.9293
**MCC**	0.8810	0.8640
**AUC**	0.9400	0.9310

The SMOTE metrics table shows the model’s performance in the training and test sets. In the training set, with a specificity of 0.9741 and sensibility of 0.9052, the model correctly identifies healthy and failing institutions. The precision (0.9722) and F1-Score (0.9375) metrics reflect a good balance between precision and sensitivity, with an MCC of 0.8810 indicating a strong correlation. Although the metrics decrease slightly in the test set, performance remains high, with specificity of 0.9608 and sensibility of 0.9020, demonstrating good generalization. The precision (0.9583) and F1-Score (0.9293) are equally robust, and the MCC (0.8640) indicates that the model maintains good predictive ability. The AUC values (0.940 in training and 0.931 in test) confirm the model’s ability to discriminate between classes in both sets.

### Construction of ARGM model

**Fig 4:**
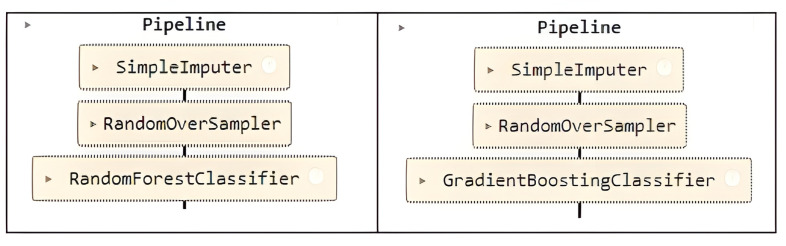
Random Forest and Gradient Boosting algorithms.

The Rough-Granular Approach [40] combines rough set theory and granular computing to handle uncertainty and complexity in data, especially in unbalanced data contexts. It partitions the feature space into granules based on similarity, improving classification in difficult problems. For the model proposed in this research, the Adjusted Rough-Granular Model (ARGM) is used, which is a simplified model using Random Forest [41] and Gradient Boosting [42], as shown in Fig 4 that applies incremental and granular learning, combining multiple “granules” or smaller models to create a global view and improve performance on unbalanced data.

**Fig 5:**
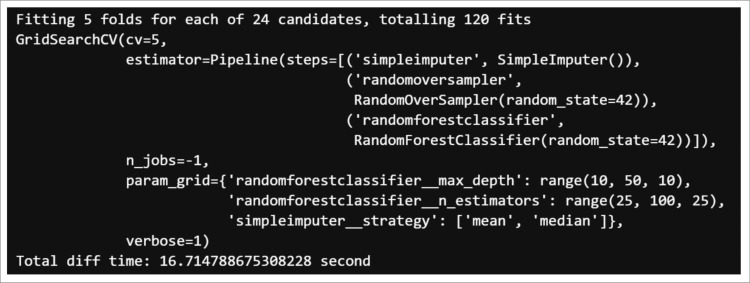
Random forest search and optimization technique.

**Fig 6:**
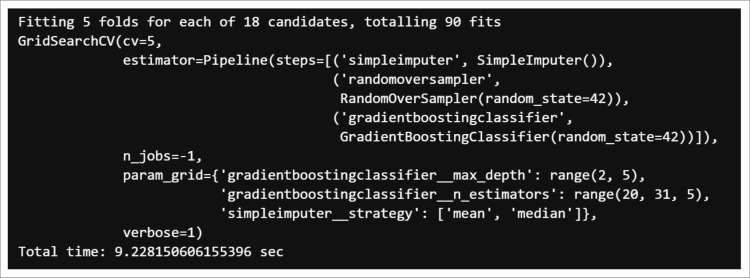
Gradient boosting search and optimization technique.

A five-step cross-validation was used. The k-fold version of the cross-validation method ensured that the percentage of samples for each class was preserved. The Python library for the GridSearchCV search and optimization technique that works with the model hyperparameters was applied Figs 5 and 6.

With the calibrated data from the previous process, we proceeded to search for the best hyperparameter scores (Accuracy) for the model and obtained values of 98.51% and 95.52%, with processing times of 15.2 and 6.41 seconds for the random forest and gradient boosting respectively. The one with the highest score was selected.

Table 8 compares the classification results of two approaches, SMOTE and ARGM (Adjusted Rough-Granular Model), evaluating their performance regarding correct and incorrect predictions for Healthy and Failure classes.

**Table 8:** Predict Class SMOTE vs ARGM.

	Predict Class SMOTE			Predict Class ARGM		
	**Healthy**	**Failure**	**Total**	**Healthy**	**Failure**	**Total**
**Healthy**	49	2	51	64	3	67
**Failure**	5	46	51	7	60	67
**Total**	54	48	102	71	63	134

The SMOTE model performs well overall, with few false positives and false negatives, handling both the majority and minority classes well after oversampling. On the other hand, the ARGM model is stronger in predicting the minority (failure) class, although with more false negatives. It is more effective in scenarios where correctly identifying failing institutions is crucial. To validate the results, several metrics were used in Table 9.

**Table 9:** SMOTE vs ARGM ranking evaluation metrics.

	SMOTE	RGCM
**Specificity**	0.9608	0.9552
**Sensibility**	0.9020	0.8955
**Precision**	0.9583	0.9524
**Accuracy**	0.9314	0.9254
**F1-Score**	0.9293	0.9231
**MCC**	0.8640	0.8520
**AUC**	0.9310	0.9250

**Fig 7:**
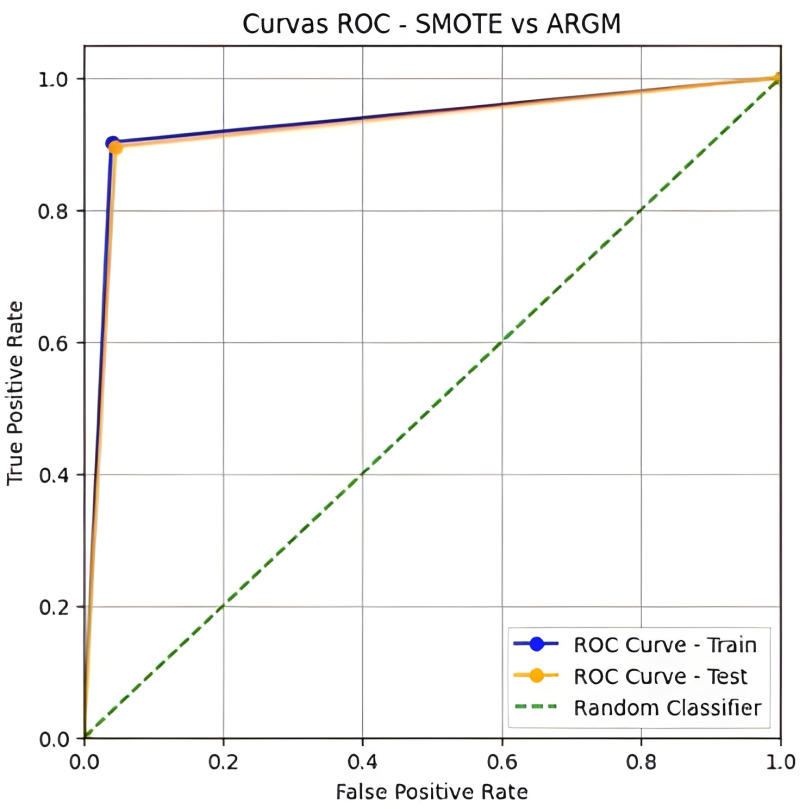
ROC curve, SMOTE versus ARGM.

As shown in Table 9, ARGM reflects a good performance of the classification model. With a specificity of 0.9552 and a sensitivity of 0.8955, the model correctly identifies healthy and failing institutions. The precision of 0.9524 and F1-Score of 0.9231 indicate a good balance between precision and sensitivity. The MCC (0.8520) and AUC (0.9250) confirm a strong correlation between predictions and actual classes and an excellent ability to discriminate between classes. Fig 7 plots the AUC for the SMOTE and ARGM.

### Theoretical and methodological implications

The Adjusted Rough-Granular Model (ARGM) significantly advances the literature on microfinance bankruptcy prediction by addressing traditional models’ critical limitations. Previous studies, such as Altman’s seminal work on Z-scores and discriminant analysis [11], and subsequent refinements using Logit and Probit models [16,19], demonstrated the importance of financial ratios in predicting bankruptcy but struggled with interpretability when extended to more complex machine learning techniques[15,27]. While recent advancements like Gradient Boosting and XGBoost [25,28] achieve high predictive accuracy, they often compromise interpretability, making them less practical for regulatory applications. By contrast, ARGM integrates granular computing principles and rough set theory to improve predictive accuracy (AUC = 0.9250) and interpretability. This dual advantage is critical in microfinance, where decision-makers require transparent models to assess risks effectively. The ARGM approach thus bridges a vital gap identified in the literature, combining state-of-the-art machine learning with practical usability, as highlighted by its robust performance even in highly imbalanced datasets.

### Failure predictions with new dataset

We prepared a new sample of 30 records, including banks, finance companies, microfinance companies, municipal microfinance, and rural savings banks. As shown in Table 10, we maintained the same financial attributes used to validate the RGCM model and predict possible institutions with failure problems.

**Table 10:** Micro financial institutions assessed with RGCM.

No	Description	Class	No	Description	Class
**1**	Bank	Healthy	**16**	Microfinance	Failure
**2**	Bank	Healthy	**17**	Rural Microfinance	Healthy
**3**	Bank	Healthy	**18**	Municipal Microfinance	Healthy
**4**	Bank	Healthy	**19**	Bank	Healthy
**5**	Municipal Microfinance	Failure	**20**	Bank	Healthy
**6**	Finance Institution	Healthy	**21**	Finance Institution	Healthy
**7**	Microfinance	Failure	**22**	Microfinance	Failure
**8**	Bank	Healthy	**23**	Bank	Healthy
**9**	Bank	Healthy	**24**	Bank	Healthy
**10**	Bank	Healthy	**25**	Microfinance	Healthy
**11**	Bank	Healthy	**26**	Microfinance	Healthy
**12**	Municipal Microfinance	Healthy	**27**	Municipal Microfinance	Healthy
**13**	Municipal Microfinance	Healthy	**28**	Finance Institution	Healthy
**14**	Municipal Microfinance	Healthy	**29**	Finance Institution	Healthy
**15**	Rural Microfinance	Failure	**30**	Microfinance	Failure

The Adjusted Rough-Granular Model, applied to evaluate 30 financial institutions with 32 financial ratio attributes validated in the previous data analysis, correctly identified that six institutions (20%) are experiencing financial health problems or failure. This indicates that the approach has effectively detected institutions at risk, crucial in financial risk prevention and management. Given the level of granularity in the prediction, the model seems to have accurately captured the entities most likely to face failure, contributing to early detection that could facilitate timely intervention. The 20/80 balance suggests that the model has a good ability to classify between failing and healthy entities.

## Conclusions and managerial implications

This study uncovered three key insights that add to our understanding of predicting failures in microfinance institutions in emerging markets. First, the methodology we developed, the Adjusted Rough-Granular Model (ARGM), proved to be a significant improvement over traditional approach. It achieved impressive results, with nearly 90% accuracy in identifying institutions at risk of failure and over 95% in spotting safe ones. This is important for a sector that relies heavily on early warning systems to mitigate financial risks and improve strategic decision making [43,44].

Also, combining SMOTE balancing techniques with granular computing gave the model a serious boost in handling the usual class imbalance that makes failure prediction tricky. What stood out to us was how well the ARGM captured the intricate patterns behind financial distress—without losing the interpretability that’s so important for practical use [43]. With an AUC score of 0.9250, this hybrid approach offers both power and clarity, making it a valuable tool for anyone working to keep microfinance institutions stable and Sustainable.

The Adjusted Rough-Granular Model (ARGM) brings something fresh and much-needed to predicting failures in microfinance institutions. For years, traditional models like Altman’s Z-scores and discriminant analysis have shown how useful financial ratios can be for this purpose. However, when these methods evolved into more complex approaches, like Logit or Probit models, and even modern machine learning techniques like Gradient Boosting and XGBoost, they faced a big challenge: interpretability. These newer models are great at making accurate predictions, but their complexity often makes it hard for decision-makers, especially regulators, to understand how the predictions are made. That’s where ARGM shines, integrating granular computing and rough set theory strikes the perfect balance between accuracy and transparency.

Predicting financial risks in microfinance institutions is difficult, especially with imbalanced datasets. That’s where the Adjusted Rough-Granular Model (ARGM) steps in. This model combines techniques like granular computing and machine learning (think Random Forest and Gradient Boosting) to identify patterns linked to financial distress.

Here’s the thing—it doesn’t just give accurate predictions; it also makes them easy to understand. In one test with a dataset of 30 institutions, the model flagged six as high-risk. That’s 20% is a big deal if you’re trying to catch problems early. What’s even better is that it helps decision-makers trust the results. For regulators and microfinance managers, they can act before things get out of hand. It’s not just a theoretical tool; it’s something people can use to make smarter decisions.

There’s a lot to unpack from our findings, especially for emerging markets. These regions face unique challenges—think about volatile economies, weaker regulatory systems, and how easily systemic shocks [1,7,8,45]. Microfinance institutions in these areas need tools to identify risks early to protect financial inclusion and avoid setbacks [2,3]. That’s where ARGM shows its value. It can spot financial distress with impressive accuracy, even when working with tricky datasets. But that’s not all. The model isn’t just accurate, it’s flexible too. It can adapt to other regions facing similar issues, like South Asia or Sub-Saharan Africa, where microfinance is essential for connecting underserved communities with financial services [5,9]. And here’s the part I find really exciting: the ARGM uses granular computing to make its predictions clear and easy to understand. This means regulators and managers can use it to design interventions that match specific regional needs; based on local financial indicators [13,21]. When you think about it, this combination of adaptability and reliability makes the ARGM more than just a theoretical framework. It’s a practical tool that can help strengthen financial stability and support economic growth in diverse emerging markets worldwide.

The model offers a valuable tool for regulators and supervisors to improve their monitoring of microfinance institutions. Using it, they could step in earlier and target interventions more effectively. Its high sensitivity means it’s less likely to miss struggling institutions. For microfinance managers, the model’s interpretability provides clear insights into which financial indicators signal trouble. This allows them to take proactive steps and address risks before becoming bigger problems. But there’s more. Our findings suggest that regulatory frameworks should be more flexible, considering the unique challenges and vulnerabilities that microfinance institutions face in emerging markets. The model could also be useful for investors, helping them evaluate the financial health of institutions with more precision. That said, there are some limitations we need to point out. The dataset we used—271 observations from 56 institutions—is relatively small, which could affect how generalizable the results are. This isn’t unusual for studies in emerging markets, where data can be hard to come by, but it’s still something to keep in mind.

While the statistical analysis demonstrates that our model achieves strong performance metrics—with sensitivity, specificity, and AUC values indicating its effectiveness—it is important to discuss the inherent trade-offs, particularly in specificity. The enhanced sensitivity achieved through SMOTE rebalancing comes with a modest decrease in specificity. This indicates that, while the model is more adept at identifying at-risk institutions (reducing false negatives), it may slightly increase the misclassification of healthy institutions (false positives). In the context of microfinance, where the cost of overlooking a failing institution can be severe, this trade-off is considered acceptable; however, it warrants further investigation to ensure balanced decision-making. Furthermore, although our dataset of 56 Peruvian financial institutions collected over nearly a decade provides valuable insights, potential biases could arise from its representativeness and the data splitting process. Despite using stratified random sampling to preserve class distributions, variations in economic conditions and institutional characteristics across different regions and time periods might influence the model’s generalizability. Future research should aim to incorporate more diverse datasets and explore alternative cross-validation strategies to further mitigate any bias introduced during data partitioning.

Another limitation of the model is its reliance solely on financial ratios, which, while objective, might miss critical aspects like governance or management effectiveness. Expanding the dataset to include more regions or adding non-financial variables could provide a more complete view of failure risks in microfinance institutions. Even so, this study offers valuable insights and practical tools for identifying risks early. The ARGM has proven reliable in handling imbalanced datasets and delivering accurate predictions. For regulators and managers, its validation with new data shows it’s more than just theory, it’s a tool with real-world potential. Future research could explore integrating macroeconomic trends and qualitative factors to make these models even more robust.

## Supporting information

S1 Appendix Financial ratios. (DOCX)

S2 Appendix Main statistics of the sample. (DOCX)

S1 Database Database used in the research. (XLSX)
