# Supplementary material for: Microfinance institutions failure prediction in emerging countries, a machine learning approach
Source: PLoS One. 2025 Apr 24;20(4):e0321989. doi: 10.1371/journal.pone.0321989 (PMC12021153; doi:10.1371/journal.pone.0321989)
Supplement: S1 Appendix — (DOCX) [file pone.0321989.s001.docx]

**S1 Appendix. Financial Ratios**

|  |  | **Previous studies** |
| --- | --- | --- |
| **C (Capital Adequacy)** | | |
| Risk-weighted assets/Total capital | CAR | Peria and Schmukler (2001), Coen et al. (2019) |
| Total debts/Share capital and reserves | TD/SCR | Ferrouhi, (2014). |
| Total equity/Total assets | TE/TA | Coen et al. (2019), Al Zaidanin, (2020). |
| Equity/ Total debts | E/TD | Al Zaidanin,(2020). |
| Reserves/ total assets | R/TA | Altman, (1968) |
|  |  |  |
| **A (Asset Quality)** | | |
| Overdue Loans/Direct Loans | OL/DL | SBS, Qualitty asset indicator |
| Loans Overdue with more than 90 days of arrears / Direct Credits | 90OL/DC | SBS, Qualitty asset indicator |
| Refinanced and Restructured Credits / Direct Credits | RRC/DC | SBS, Qualitty asset indicator |
| Overdue Credits MN / Direct Credits MN | OCMN/DCMN | SBS, Qualitty asset indicator |
| Overdue Credits ME / Direct Credits ME | OCME/DCME | SBS, Qualitty asset indicator |
| Provisions / Overdue Credits | P/DC | SBS, Qualitty asset indicator |
| High Risk Portfolio / Direct Credits (%) | HRP/DC | SBS, Qualitty asset indicator |
| Provisions Expense/ Financial income | PE/FI | SBS, indicator of qualitty asset |
| Provisions / Total portfolio (current + overdue + refinanced) | P/TP | Coen et al. (2019), Ferrouhi, (2014), Hadad et al, 2011, Al Zaidanin,(2020). |
| Raw portfolio (current + overdue + refinanced)/ total assets | RP/TA | Loans high risk vs total asset |
| Net property, furniture and equipment/ Total assets | NPPE/TA | More assets in case of bankrupcy, dont exposed at risk |
| Total debts/ Total assets | TD/TA | Al Zaidanin,(2020). |
| **M (Management Quality)** | | |
| Administration Expenses / Productive Assets | AE/PA | Peria and Schmukler (2001) |
| Operating Expenses / Total Financial Margin | OE/MF | Adaptation from CAMEL model for the paper purpose |
| Financial Income / Total Income | FI/TI | Al Zaidanin,(2020). |
| Financial Income / Productive Assets | FI/PA | Al Zaidanin,(2020). |
| Direct Loans / Personnel | DL/P | Adaptation from CAMEL model for the paper purpose |
| Deposits / Number of Offices | D/NO | SBS, Management quality indicator |
| Deposits / Direct Loans (%) | D/DL | SBS, Management quality indicator |
| Financial expenses/ Financial income | FE/FI | Coen et al. (2019) |
| Public deposits / Raw portfolio | PD/RP | Adaptation from CAMEL model for the paper purpose |
| Financial income /Total assets | FI/TA | Al Zaidanin,(2020). |
| **E (Earnings)** | | |
| Net Income / Equity | NI/E | Al Zaidanin,(2020). |
| Net Income / Assets | NI/A | Al Zaidanin,(2020). |
| Net operating margin/Total Assets | NOM/TA | Coen et al. (2019) |
| Net profit/ Financial income | NP/FI | Adaptation from CAMEL model for the paper purpose |
| **L (Liquidity)** | | |
| Liquidity Ratio MN | LRMN | SBS, Liquidity indicator |
| Liquidity Ratio ME | LRME | SBS, Liquidity indicator |
| Cash and Banks MN / Demand Obligations MN | CBMN/DOMN | SBS, Liquidity indicator |
| Cash and Banks in ME / Demand Obligations ME | CBME/DOME | SBS, Liquidity indicator |
| Loans short term / Total Loans (%) | LST/TL | SBS, Liquidity indicator |
| Available funds/(Sight deposits + savings) not fixed term | AF/D | Hadad et al, 2011 |
| Public deposits/ total assets | PD/TA | Ferrouhi, (2014). |
