# Supplementary material for: Microfinance institutions failure prediction in emerging countries, a machine learning approach
Source: PLoS One. 2025 Apr 24;20(4):e0321989. doi: 10.1371/journal.pone.0321989 (PMC12021153; doi:10.1371/journal.pone.0321989)
Supplement: S2 Appendix — (DOCX) [file pone.0321989.s002.docx]

**S3 Appendix. Statistics of the full sample of 32 financial rates.**

|  | **Attr1** | **Attr2** | **Attr3** | **Attr4** | **Attr5** | **Attr6** | **Attr7** | **Attr8** |
| --- | --- | --- | --- | --- | --- | --- | --- | --- |
| **Mean** | 0.82 | 0.18 | 0.68 | 0.20 | 0.10 | 0.68 | 0.80 | 0.89 |
| **Median** | 0.85 | 0.15 | 0.77 | 0.18 | 0.09 | 0.74 | 0.80 | 0.92 |
| **Mode** | 0.11 | 0.06 | -23.57 | 0.00 | 0.01 | 0.00 | 0.33 | 0.00 |
| **SD** | 0.13 | 0.13 | 1.49 | 0.12 | 0.05 | 0.18 | 0.09 | 0.27 |
| **Maximun** | 0.94 | 0.89 | 1.00 | 0.82 | 0.60 | 0.88 | 1.06 | 1.61 |
| **Minimun** | 0.11 | 0.06 | -23.57 | -0.06 | 0.01 | 0.00 | 0.33 | 0.00 |
| **IQR (Q3-Q1)** | 0.06 | 0.06 | 0.08 | 0.14 | 0.04 | 0.16 | 0.10 | 0.27 |
| **25%** | 0.81 | 0.13 | 0.73 | 0.12 | 0.07 | 0.63 | 0.75 | 0.77 |
| **50%** | 0.85 | 0.15 | 0.77 | 0.18 | 0.09 | 0.74 | 0.80 | 0.92 |
| **75%** | 0.87 | 0.19 | 0.80 | 0.26 | 0.11 | 0.79 | 0.85 | 1.03 |
| **Skewness** | Asym.  (-) | Asym.  (+) | Sym. | Sym. | Sym. | Sym | Sym. | Sym. |

|  | **Attr9** | **Attr10** | **Attr11** | **Attr12** | **Attr13** | **Attr14** | **Attr15** | **Attr16** |
| --- | --- | --- | --- | --- | --- | --- | --- | --- |
| **Mean** | 0.03 | 0.34 | 0.03 | 0.02 | 0.18 | -0.62 | 0.32 | 19.88 |
| **Median** | 0.02 | 0.18 | 0.01 | 0.02 | 0.17 | 0.04 | 0.23 | 15.05 |
| **Mode** | 0.00 | 0.07 | 0.00 | 0.00 | 0.00 | -158.65 | 0.00 | 14.08 |
| **SD** | 0.02 | 0.80 | 0.04 | 0.11 | 0.08 | 9.66 | 1.49 | 24.66 |
| **Maximun** | 0.24 | 8.02 | 0.23 | 1.71 | 0.49 | 1.70 | 24.57 | 196.53 |
| **Minimun** | 0.00 | 0.07 | 0.00 | -0.18 | 0.00 | -158.65 | 0.00 | 6.06 |
| **IQR (Q3-Q1)** | 0.02 | 0.09 | 0.02 | 0.03 | 0.07 | 0.13 | 0.08 | 3.32 |
| **25%** | 0.01 | 0.14 | 0.01 | 0.00 | 0.15 | -0.03 | 0.20 | 13.72 |
| **50%** | 0.02 | 0.18 | 0.01 | 0.02 | 0.17 | 0.04 | 0.23 | 15.05 |
| **75%** | 0.04 | 0.23 | 0.03 | 0.03 | 0.21 | 0.10 | 0.27 | 17.04 |
| **Skewness** | Sym. | Asym.  (+) | Sym | Sym. | Asym.  (+) | Asym.  (-) | Sym. | Asym.  (+) |

|  | **Attr17** | **Attr18** | **Attr19** | **Attr20** | **Attr21** | **Attr22** | **Attr23** | **Attr24** |
| --- | --- | --- | --- | --- | --- | --- | --- | --- |
| **Mean** | 5.59 | 7.67 | 6.43 | 8.24 | 145.36 | 10.71 | 12.64 | 77.82 |
| **Median** | 5.38 | 6.20 | 4.94 | 6.48 | 134.13 | 8.90 | 11.69 | 68.19 |
| **Mode** | 5.45 | 5.32 | 1.88 | 6.56 | 104.35 | 5.55 | 13.21 | 69.50 |
| **SD** | 2.84 | 5.53 | 5.50 | 6.22 | 54.91 | 6.74 | 5.29 | 40.48 |
| **Maximun** | 29.64 | 57.62 | 57.57 | 57.62 | 425.85 | 58.14 | 55.83 | 415.08 |
| **Minimun** | 0.09 | 0.00 | 1.30 | 0.00 | 21.64 | 3.31 | 2.49 | 24.29 |
| **IQR (Q3-Q1)** | 3.34 | 3.92 | 3.55 | 4.89 | 52.99 | 5.77 | 5.96 | 21.33 |
| **25%** | 3.93 | 4.55 | 3.37 | 4.51 | 110.87 | 6.44 | 9.22 | 59.97 |
| **50%** | 5.38 | 6.20 | 4.94 | 6.48 | 134.13 | 8.90 | 11.69 | 68.19 |
| **75%** | 7.26 | 8.47 | 6.92 | 9.39 | 163.86 | 12.21 | 15.19 | 81.30 |
| **Skewness** | Asym.  (+). | Sym. | Sym. | Sym. | Sym. | Sym. | Sym. | Sym. |

|  | **Attr25** | **Attr26** | **Attr27** | **Attr28** | **Attr29** | **Attr30** | **Attr31** | **Attr32** |
| --- | --- | --- | --- | --- | --- | --- | --- | --- |
| **Mean** | 21.83 | 976.45 | 17363.85 | 99.97 | 0.49 | 0.28 | 39.14 | 392.77 |
| **Median** | 19.89 | 841.33 | 12880.68 | 98.15 | 4.50 | 0.62 | 27.88 | 96.96 |
| **Mode** | 24.20 | 314.00 | 234.48 | 94.11 | -113.02 | 1.27 | 23.62 | 0.00 |
| **SD** | 8.29 | 744.20 | 14423.52 | 18.63 | 19.64 | 3.26 | 69.85 | 1383.99 |
| **Maximun** | 53.41 | 5324.00 | 170159.44 | 161.06 | 36.97 | 9.16 | 961.72 | 12655.63 |
| **Minimun** | 0.66 | 0.00 | 234.48 | 22.94 | -113.02 | -25.66 | 9.49 | 0.00 |
| **IQR (Q3-Q1)** | 7.45 | 621.01 | 15011.35 | 18.30 | 17.43 | 2.92 | 19.14 | 65.79 |
| **25%** | 17.57 | 534.39 | 8922.79 | 90.14 | -5.05 | -0.96 | 20.47 | 69.58 |
| **50%** | 19.89 | 841.33 | 12880.68 | 98.15 | 4.50 | 0.62 | 27.88 | 96.96 |
| **75%** | 25.02 | 1155.40 | 23934.14 | 108.44 | 12.38 | 1.96 | 39.61 | 135.36 |
| **Skewness** | Sym. | Sym. | Sym. | Sym. | Sym. | Sym. | Sym. | Sym. |
